# Information Theory and Its Application in Machine Condition Monitoring

**DOI:** 10.3390/e24020206

**Published:** 2022-01-28

**Authors:** Yongbo Li, Fengshou Gu, Xihui Liang

**Affiliations:** 1 School of Aeronautics, Northwestern Polytechnical University, Xi’an 710072, China; 2 Centre for Efficiency and Performance Engineering (CEPE), University of Huddersfield, Queensgate, Huddersfield HD1 3DH, UK; F.Gu@hud.ac.uk; 3 Department of Mechanical Engineering, University of Manitoba, Winnipeg, MB R3T 5V6, Canada; xihui.liang@umanitoba.ca

## Introduction

Rotating machinery is part and parcel of modern industrial applications. Due to their continuous nature of operation in harsh and varying operating conditions, rotating machinery is much more prone to failure. Failure to diagnose the fault on time can lead to catastrophic effects from both a financial and safety point of view. In this context, research on entropy theory and its application in machine condition monitoring is very interesting, as it can bring some new insights into the prognostic and health management of academic and industrial files. Information theory, as one of the greatest discoveries in the history of science, has formed more than ten different calculation forms after more than 70 years of development. Information theory has been widely used in the feature extraction field due to its merits of independence with prior knowledge, the lack of a need to preprocess, and ease of performance. It has powerful ability for feature representation, has received extensive attention from both academic and industrial attentions.

This Special Issue in Entropy aims to collect recent research results and the latest developments in condition monitoring techniques by means of information theory. This collection contains 10 papers that represent the state of the art application of information theory in the field of condition monitoring.

To resolve the dilemma of diagnosing non-structural failures, Mao et al. proposed a fusion domain-adaptation convolutional neural network (FDACNN) to combine infrared thermal images and vibration signals for the diagnosis of both structural and non-structural failures under various working conditions [1]. They designed an adversarial network to recognize of the health condition of structural and non-structural faults in the unlabeled target domain. The results suggest that the proposed FDACNN method performs best in the cross-domain fault diagnosis of gearboxes via multi-source heterogeneous data.

Bai et al. proposed a new method based on an improved YOLOv4 for railway surface defect detection [2]. In this method, MobileNetv3 was used as the backbone network of YOLOv4 to extract image features, and deep separable convolution as applied on the PANet layer in YOLOv4 to realize the lightweight network and achieve real-time detection of the railway surface.

Xiao et al. diagnosed the different types of misalignment fault, such as parallel misalignment, angular misalignment and integrated misalignment, of a wind turbine by means of information fusion [3]. The concept of information fusion was realized by fusing different types of sensor data, such as vibration, temperature and current. Finally, fault diagnosis of the wind turbine was achieved from the fused information with the help of Dempster–Shafer evidence theory.

Jia et al. showed a partial transfer fault diagnosis model based on a weighted subdomain adaptation network (WSAN) [4]. This model paid more attention to the important local data distribution, while aligning the global distribution through a specially designed weighted local maximum mean discrepancy (WLMMD), which was able to align relevant distributions of domain-specific layer activations across different domains.

Targeting the challenges regarding weak fault signal, coupling among different dimensions of the collected signal, and scarcity of fault datasets, Wei et al. proposed a novel fault detection based on multi-dimensional KDE and Jensen–Shannon divergence [5]. Addressing the limitations of the conventional KDE method regarding information loss for multidimensional problems, in this research, it was extended to a multidimensional version for tackling the weak fault signal and coupling problem of the collected signal. Later, Jensen–Shannon divergence was used for unknown fault detection.

Hu et al. proposed a subway gearbox fault diagnosis algorithm based on adaptive spline impact suppression [6]. Signals collected from the gearbox in a subway are often associated with the impacts between wheelsets and rail joint gaps. A long time-series is more often affected by unsteadiness due to the wheel-rail impacts. Motivated by this phenomenon, in this paper, long times-series were segmented adaptively into short time series, in order to suppress the effect of the high amplitude of the shock response signal. The adaptive segmentation of the original signal was achieved using a cubic spline interpolation algorithm. The proposed method was verified by data collected from a real Beijing subway line.

Yan et al. proposed a universal domain adaptation method to recognize unknown fault types in the target domain [7]. They took into account the discrepancy of the fault features shown by different fault types, and formed the feature center for fault diagnosis by extracting the features of the samples of each fault type.

Wind turbines are one of the major sources of green energy. However, the majority of the wind turbine fault diagnosis research is focused on single faults rather than combined faults. In order to fill this gap, in this Special Issue, Xiao et al. proposed a machine-learning-based approach with the help of the feature extracted by a novel low-pass filtering empirical wavelet transform [8].

Li et al. proposed a novel anomaly detection framework for a satellite momentum wheel [9]. Aimed at the lack of research on simulation data, and the scarcity of research on real telemetry data, the proposed framework was able to detect anomalies in a satellite momentum wheel, based on the features extracted by a newly proposed Huffman-multi-scale entropy method. The extracted features were then classified by an optimized support vector machine algorithm.

Mao et al. suggested a new online detection method, called the deep dual-temporal domain adaptation model (DTDA), to effectively extract a domain-invariant temporal feature representation for online early-fault detection of bearings [10]. They developed a new state assessment method to determine the period of the normal state and that of the degradation state for whole-life degradation sequences. A health indicator of target bearings was also built based on the DTDA features, to intuitively evaluate the detection results.

The editors extend their heartiest gratitude toward all of the the authors for their excellent contribution to this Special Issue. Special thanks to all of the anonymous reviewers for their time and feedback provided to the authors. Additionally, we sincerely thank the publishers, editors and all of the members of the Entropy editorial board for facilitating this opportunity to present all of the works.

## References

[1] Mao G., Zhang Z., Qiao B., Li Y. (2022). Fusion domain-adaptation CNN driven by images and vibration signals for fault diagnosis of gearbox cross-working conditions. Entropy.

[2] Bai T., Gao J., Yang J., Yao D. (2021). A study on railway surface defects detection based on machine vision. Entropy.

[3] Xiao Y., Xue J., Zhang L., Wang Y., Li M. (2021). Misalignment fault diagnosis for wind turbines based on information fusion. Entropy.

[4] Jia S., Wang J., Zhang X., Han B. (2021). A weighted subdomain adaptation network for partial transfer fault diagnosis of rotating machinery. Entropy.

[5] Wei J., He Z., Wang J., Wang D., Zhou X. (2021). Fault detection based on multi-dimensional KDE and Jensen–Shannon divergence. Entropy.

[6] Hu Z., Yang J., Yao D., Wang J., Bai Y. (2021). Subway gearbox fault diagnosis algorithm based on adaptive spline impact suppression. Entropy.

[7] Yan Z., Liu G., Wang J., Bao H., Zhang Z., Zhang X., Han B. (2021). A new universal domain adaptive method for diagnosing unknown bearing faults. Entropy.

[8] Xiao Y., Xue J., Li M., Yang W. (2021). Low-pass filtering empirical wavelet transform machine learning based fault diagnosis for combined fault of wind turbines. Entropy.

[9] Li Y., Lei M., Liu P., Wang R., Xu M. (2021). A novel framework for anomaly detection for satellite momentum wheel based on optimized SVM and Huffman-Multi-Scale entropy. Entropy.

[10] Mao W., Sun B., Wang L. (2021). A new deep dual temporal domain adaptation method for online detection of bearings early fault. Entropy.

